# Patient expectations for placebo treatments commonly used in osteopathic manipulative treatment (OMT) clinical trials: a pilot study

**DOI:** 10.1186/1750-4732-1-3

**Published:** 2007-01-12

**Authors:** Kimberly G Fulda, Turner Slicho, Scott T Stoll

**Affiliations:** 1 Osteopathic Research Center, University of North Texas Health Science Center, 3500 Camp Bowie Blvd, Fort Worth, TX 76107, USA

## Abstract

**Background:**

Placebo treatments should be believable to ensure expectation of benefit, yet not provide a true treatment effect. One obstacle to conducting clinical trials with osteopathic manipulative treatment (OMT) is choosing an appropriate placebo. Various placebo treatments have been used in OMT clinical trials. The purpose of this study was to determine expectations of 3 treatments (HVLA, placebo light touch, placebo sub-therapeutic ultrasound) commonly used in OMT clinical research trials.

**Methods:**

A randomized, cross-over design was utilized. Subjects were recruited from the Family Medicine Clinic, Texas College of Osteopathic Medicine. Participants watched a video with 2 minute demonstrations of a High Velocity Low Amplitude (HVLA), placebo light touch (LT), and placebo sub-therapeutic ultrasound (ULTRA) treatment for low back pain. The order of presentations was randomized to control for order effect bias. Subjects indicated the extent of their agreement (using a 4 point Likert scale) with 4 statements that were presented after each treatment was viewed: 1)I believe this treatment would allow me to get better quicker; 2)I believe this treatment would decrease my low back pain; 3)I believe this treatment would make me more able to do the things I want to do; 4)This seems like a logical way to treat low back pain. Repeated measures analysis of variance was performed, and a partial Eta squared was calculated for each statement. Effect sizes (Cohen's d) were calculated where appropriate.

**Results:**

Thirty of 40 eligible subjects participated. Twenty-two (73%) were female, 16 (53%) were Caucasian, and 11 (37%) had completed college. The mean age was 43 (SD = 15.). Repeated measures ANOVA revealed no significant differences for statements 2 and 4. For both statements 1 (p = 0.025) and 3 (p = 0.039), post hoc analysis revealed a difference between HVLA and LT. The partial Eta squared (η_p_^2^) was 0.105, 0.072, 0.107, and 0.024 for each statement, respectively.

**Conclusion:**

There is a difference in treatment expectation between HVLA and LT for statements 1 and 3. Participants responded more positively after viewing the HVLA treatment than the LT treatment. This suggests that sub-therapeutic ultrasound is the better placebo because the expectations were similar to those for HVLA.

## Background

Randomized controlled trials (RCTs) are considered the most rigorous form of research and are commonly used to evaluate the effectiveness of medical interventions. An RCT compares an intervention to either a placebo, gold standard, or both. Selecting the optimal placebo group is essential for valid comparison between groups and to measure the effectiveness of an intervention. A placebo treatment should be believable to ensure expectation of benefit, yet not provide a true treatment effect [1,2]. Thus, to a subject in a pharmaceutical clinical trial, the placebo treatment should appear indistinguishable from the active treatment.

Several definitions have been used to define placebo. The strictest traditional interpretation of the definition would limit the use of a placebo to only pharmaceutical trials using sugar pills as the placebo treatment. Such a definition is not useful in clinical trials of non-pharmaceutical interventions such as manipulative treatment. One definition of placebo that attempts to incorporate non-pharmaceutical interventions is "a substance or procedure that has no inherent power to produce an effect that is sought or expected" [3]. McQuay and Moore more broadly define placebo effect as "the effect that we observe when patients are given a placebo," or "the effect caused by placebo" [4]. Hróbjartsson suggests there are three main elements of placebo effect including: change after placebo medication (pre/post change in placebo group), effect of placebo intervention (treatment experience), and effect of patient – provider interaction [5]. The term placebo response is often used interchangeably with placebo effect.

There are two prominent theories for why the placebo effect exists. The conditioning theory suggests that when pairing a neutral stimulus with an unconditioned stimulus (such as the active drug) the neutral stimulus elicits a response, resulting in a conditioned response [6]. In contrast, the expectancy theory is based on the patient expectations. The response to a stimulus depends on what response is expected from the stimulus. These patient expectations may not account for all of the placebo effect, but they are the most significant factor of the expectancy theory [3].

A common misconception about placebos is that a third of the population will demonstrate a placebo response. This is not always the case. McQuay and Moore found that placebo response varies from 7% for pain treatment of migraines to 49% for pain treatment of diabetic neuropathy [4]. The response to placebos in clinical research trials differs according to the length of the trial, the medical condition studied, the placebo used, and various other factors [7]. Patient-physician interaction plays a role in placebo response. For example, a physician's attitude (enthusiastic versus doubtful) towards an intervention can significantly influence a patient's health outcomes such as pain, psychiatric illness, hypertension, and obesity [8].

The informed consent process also affects the placebo response. In one clinical trial, cancer patients not needing major analgesics were given either naproxen or a placebo for pain. Some of the patients were informed that they were participating in a clinical trial and would receive either naproxen or a placebo, while others were not informed about the trial. The patients who were informed of the possibility of receiving a placebo and actually received the placebo had greater pain relief than those patients who were not informed of the trial and were given naproxen [9]. Additionally, the active drug to placebo treatment ratio of a trial may affect placebo response. Diener et al. examined the use of placebo in migraine trials. They found that participants in clinical trials with a greater active drug to placebo treatment ratio had a greater placebo response due to a higher expectation of receiving treatment [10].

Choosing an appropriate placebo treatment, sometimes referred to as ~sham~treatment, is an obstacle when conducting clinical trials with manipulative treatment. It is difficult to develop a placebo that mimics osteopathic manipulative treatment (OMT) or chiropractic treatment and produces the expectation of benefit. A variety of placebo treatments such as light touch [11], sham manipulation [12], and sub-therapeutic ultrasound [13] have been used in manual therapy clinical trials. Vernon et al. reported that after receiving a cervical sham manipulation, study participants did not report clinically significant changes in range of motion or tenderness. This study, however, included 20 subjects of which only three had no previous experience with chiropractic treatment [14]. Hawk et al. reported improvement in subjects with subacute or chronic low back pain in both placebo and active manipulation groups. There were no differences in the amount of improvement between the groups, even after controlling for prior chiropractic experience and initial treatment expectations [15]. Furthermore, experts do not necessarily agree on what constitutes the active component of a treatment or placebo. In a recent study, a list of 10 placebo manipulative techniques (including a description of each) was developed and sent to experts in Australia and New Zealand. At least one of the 16 respondents replied that each technique had an active component, and none of the techniques were considered an appropriate placebo by at least 50% of the respondents [16]. To determine the credibility of light touch and sub-therapeutic ultrasound as compared to High-Velocity Low-Amplitude (HVLA) and standard of care, Slicho conducted a survey of the general population. Survey respondents more strongly agreed with HVLA as a way to logically treat low back pain, but responses did not differ in other aspects of treatment expectation after reading a description of the treatments [17].

Understanding the placebo effect and developing the best placebo is vital to studying the effectiveness of OMT. This current study was designed as a continuation of the research conducted by Slicho [17]. Clinic patients were asked to view a video of three types of treatments (one active and two placebos) for chronic low back pain, and responses for expectation of benefit from the treatments were measured. The primary purpose of this research study was to determine attitudes towards different types of treatments commonly used in OMT clinical research trials. A secondary question was whether or not these attitudes are different if a person has previously received OMT or chiropractic treatment.

## Methods

This pilot study utilized a randomized, cross-over design. Subjects 18 years of age and older were recruited from the Family Medicine Clinic of the Texas College of Osteopathic Medicine in Fort Worth, TX during February through May 2005. Subjects were asked to participate in the study while waiting for their regularly scheduled physician appointment. They were approached if their participation would not interfere with clinic operations.

Participants were asked to watch a video with 2 minute demonstrations representing a High-Velocity Low-Amplitude (HVLA), placebo light touch (LT), and placebo sub-therapeutic ultrasound (ULTRA) treatment for low back pain. The HVLA treatment consisted of lateral recumbent lumbar soft tissue treatment followed by a 'lumbar roll' manipulation applied to both the left and right side of the simulated patient. The LT treatment consisted of a series of static bilateral hand placements on the posterior thoraco lumbar junction and upper pelvis followed by anterior lower ribs and pelvis of the simulated patient. The ULTRA treatment consisted of a close up image of the ultrasound device followed by sequential application of the non-functional ultrasound applicator (without gel) in circular motions with mild pressure to the same anterior and posterior bilateral body areas on the simulated patient to which the light touch was applied. All treatments were videotaped on the same day using the same professionally dressed osteopathic physician and same casually dressed simulated female patient. All treatments were applied through the clothing. Treatments were applied for 2 minutes with 1 minute allocated for each side. All treatments required the simulated patient to lie down, change sides, and return to a sitting position. The simulated patient began and ended each treatment in the sitting position facing the camera with the physician standing behind her.

The order in which subjects viewed the demonstrations was randomized to control for order effect bias. Six video tapes were available to account for the different combinations of treatments. The combinations were HVLA, LT, ULTRA; HVLA, ULTRA, LT; ULTRA, HVLA, LT; ULTRA, LT, HVLA; LT, HVLA, ULTRA; and LT, ULTRA, HVLA. A verbal description of each treatment narrated by the same person was included on the video, and a written description was included on each survey. Subjects indicated the extent of their agreement (using a 4 point Likert scale) with 4 statements that were presented after each treatment was viewed: 1. "I believe this treatment would allow me to get better quicker"; 2. "I believe this treatment would decrease my low back pain"; 3. "I believe this treatment would make me more able to do the things I want to do"; 4. "This seems like a logical way to treat low back pain" (Table 1). Demographic information and previous experience with osteopathic manipulative treatment, chiropractic treatment, massage therapy, and ultrasound were collected; however, a distinction was not made between previous experience with diagnostic and therapeutic ultrasound.

**Table 1:** Survey Statements*

S1	I believe this treatment would allow me to get better quicker.
S2	I believe this treatment would decrease my low back pain.
S3	I believe this treatment would make me more able to do the things I want to do.
S4	This seems like a logical way to treat low back pain.

* Responses were 1 = Strongly Agree, 2 = Agree, 3 = Disagree, 4 = Strongly Disagree

### Statistical analysis

Repeated measures analysis of variance (ANOVA) was performed on each of the 4 questions to determine differences in responses for treatments. Post hoc analyses were performed using Least Significant Difference (LSD). LSD was used because of the pilot nature of the research study. It does not adjust for the number of comparisons in the post hoc analyses. A partial Eta squared (η^2^_p_) was calculated for each of the 4 statements, representing the proportion of the total variability accounted for by the test. A Cohen's d (*d*) was calculated for the different possible combinations of the four treatment presentations. Cohen's d is an effect size calculated as (mean of treatment group 1 – mean of treatment group 2)/the pooled standard deviation. Throughout this manuscript, treatment group 1 and treatment group 2 are ordered as HVLA and ULTRA; ULTRA and LT; or HVLA and LT. Cohen's d values are considered to be a small effect size at 0.2, a moderate effect size at 0.5, and a large effect size at 0.8 [18].

Participants responding "yes" to the question "Have you ever had osteopathic manipulative treatment" or "Have you ever had chiropractic treatment" were combined since small numbers of respondents prevented meaningful separation for sub group analysis. Independent samples t tests were performed for each question relating to the ULTRA and LT groups to determine differences between those having ever had manipulative treatment (osteopathic or chiropractic) and those not having ever had manipulative treatment.

Results were considered significant at the alpha 0.05 level. All analyses were performed using SPSS 12.0 (SPSS Inc, Chicago, IL). Study procedures were approved by the University of North Texas Health Science Center's Institutional Review Board.

## Results

Thirty of 40 eligible subjects participated for a 75% response rate. Demographic characteristics of the sample are presented in Table 2. Of the participants, 22 (73%) were female, 16 (53%) were Caucasian, and 11 (37%) had completed college. The mean age was 43 years (SD = 15, min = 20, max = 68). Ten (33.3%) subjects had previously experienced osteopathic manipulative treatment, 14 (46.7%) chiropractic treatment, 11 (36.7%) massage therapy, and 20 (66.7%) ultrasound. Mean responses for each question are presented in Table 3. The sphericity assumption for repeated measures ANOVA was met for all questions.

**Table 2:** Demographic characteristics of sample

	n	%
Gender		
Male	8	26.7
Female	22	73.3
Race/Ethnicity		
Caucasian	16	53.3
Hispanic	7	23.3
Other	7	23.3
Education		
Some High School	5	16.7
High School	4	13.3
Some College	10	33.3
College	11	36.6
Ever Had Massage Therapy		
Yes	11	36.7
No	19	63.3
Ever Had Osteopathic Manipulative Treatment		
Yes	10	33.3
No	20	66.7
Ever Had Chiropractic Treatment		
Yes	14	46.7
No	16	53.3
Ever Had Ultrasound		
Yes	20	66.7
No	10	33.3

**Table 3:** Mean responses to questions*

	HVLA		ULTRA		LT	
	mean (SD)	95% CI	mean (SD)	95% CI	mean (SD)	95% CI

S1	1.97 (0.67)	(1.72, 2.22)	2.17 (0.70)	(1.91, 2.43)	2.33 (0.80)	(2.03, 2.63)
S2	1.93 (0.69)	(1.68, 2.19)	2.00 (0.59)	(1.78, 2.22)	2.20 (0.71)	(1.93, 2.47)
S3	1.93 (0.79)	(1.64, 2.23)	2.23 (0.68)	(1.98, 2.49)	2.30 (0.79)	(2.00, 2.60)
S4	1.97 (0.81)	(1.66, 2.27)	2.13 (0.73)	(1.86, 2.41)	2.13 (0.86)	(1.81, 2.45)

*SD = Standard Deviation; HVLA – High-Velocity Low-Amplitude; ULTRA = Sub-therapeutic Ultrasound; LT = Light Touch

Statement 1: "I believe this treatment would allow me to get better quicker"

Repeated measures analyses of variance (ANOVA) revealed an overall statistically significant difference in responses between groups for Statement 1, (F(2,58) = 3.388, p = 0.041) (Table 4). Post hoc analysis identified significant differences in responses between HVLA and LT (p = 0.025) with a mean difference of -0.367 out of a maximum of 4. Participants responded more positively to the statement "I believe this treatment would allow me to get better quicker" for the HVLA treatment. There were no statistically significant differences between responses for HVLA and ULTRA (p = 0.136) or between ULTRA and LT (p = 0.231). The partial eta squared (η^2^_p_) = 0.105.

**Table 4:** Repeated measures ANOVA and partial Eta squared*

	F	P	η^2^_p_
S1	3.388	0.041	0.105
S2	2.257	0.114	0.072
S3	3.485	0.037	0.107
S4	0.707	0.497	0.024

*P = p value; degrees of freedom = (2, 58); η^2^_p _= Partial Eta Squared

Cohen's d (*d*) = -0.29 for HVLA and ULTRA; -0.21 for ULTRA and LT; and -0.49 for HVLA and LT (Table 5). Each of these represents a medium effect size between responses for the treatment groups. The Cohen's d represents a borderline medium effect size for HVLA and ULTRA and for ULTRA and LT; however, the there is a borderline medium/large effect size for HVLA and LT.

**Table 5:** Effect Sizes (Cohen's d) for Each Statement for Treatment Groupings*

	HVLA & ULTRA	ULTRA & LT	HVLA & LT
S1	-0.29	-0.21	-0.49
S2	-0.11	-0.31	-0.38
S3	-0.41	-0.09	-0.47
S4	-0.21	0	-0.19

*HVLA – High-Velocity Low-Amplitude; ULTRA = Sub-therapeutic Ultrasound;

LT = Light Touch.

Cohen's d – 0.2 small effect; 0.5 moderate effect; 0.8 large effect [18].

Statement 2: "I believe this treatment would decrease my low back pain"

Repeated measures ANOVA revealed no statistically significant difference between responses to Statement 2, "I believe this treatment would decrease my low back pain", (F(2,58) = 2.257, p = 0.114) (Table 4). The partial eta squared (η^2^_p_) = 0.072.

Cohen's d (*d*) = -0.11 for HVLA and ULTRA; -0.31 for ULTRA and LT; and -0.38 for HVLA and LT (Table 5). A small effect is observed for HVLA and ULTRA. A medium effect is observed for both ULTRA and LT; and HVLA and LT, suggesting similar responses for each group.

Statement 3: "I believe this treatment would make me more able to do the things I want to do"

Statistically significant differences were found between responses for Statement 3, (F(2,58) = 3.485, p = 0.037) with repeated measures ANOVA (Table 4). Post hoc analysis demonstrated significant differences between HVLA and LT (p = 0.039) with a mean difference of -0.367 and between HVLA and ULTRA (p = 0.048) with a mean difference of -0.300. Participants responded more positively to the statement "I believe this treatment would make me more able to do the things I want to do" for HVLA than both the LT and ULTRA. There was not a statistically significant difference between ULTRA and LT (p = 0.601). The partial eta squared (η^2^_p_) = 0.107.

Cohen's d (*d*) = -0.41 for HVLA and ULTRA; -0.09 for ULTRA and LT; and -0.47 for HVLA and LT (Table 5). A very small effect is observed for ULTRA and LT, while a medium effect is observed for HVLA and ULTRA; and HVLA and LT. This suggests similar responses for HVLA and ULTRA and for HVLA and LT.

Statement 4: "This seems like a logical way to treat low back pain"

Repeated measures ANOVA revealed no statistically significant difference between responses to Statement 4, "This seems like a logical way to treat low back pain", (F(2,58) = 0.707, p = 0.497) (Table 4). The partial eta squared (η^2^_p_) = 0.024.

Cohen's d (*d*) = -0.21 for HVLA and ULTRA; 0 for ULTRA and LT; and -0.19 for HVLA and LT (Table 5). There is no effect observed for ULTRA and LT for this question and only a small effect for HVLA and LT. A marginally medium effect is observed for HVLA and ULTRA.

### Manipulative treatment

A significant difference was observed between those having ever received manipulative treatment (osteopathic manipulative treatment or chiropractic treatment) and those not having ever received manipulative treatment when responding to Statement 1 for the ULTRA group (p = 0.03). Those who had never received manipulative treatment (mean = 1.83) responded more favourably than those who had received manipulative treatment (mean = 2.39).

No other differences were found between having ever received manipulative treatment and not having ever received manipulative treatment for any other question pertaining to either the ULTRA or LT treatments. However, the difference did approach significance for Statement 4 with the ULTRA treatment (p = 0.06), also with those who had never received manipulative treatment (mean = 1.83) responding more favourably than those who had received manipulative treatment (mean = 2.33).

A similar analysis of subjects who had never received ultrasound and subjects who had received ultrasound was conducted. No significant differences were observed between the groups for any of the 4 statements relating to HVLA or LT.

## Discussion

This study aimed to measure treatment expectations of patients in a Family Medicine clinic after watching a video with short demonstrations of one active and two placebo treatments that have all been used in previous clinical trials of osteopathic manipulative treatment (OMT). In a previous study, Slicho provided information on treatment expectations of the general population measured through a postal survey with descriptions representing High-Velocity Low-Amplitude (HVLA), sub-therapeutic ultrasound, light touch, and standard care. Slicho found that study participants more strongly agreed with HVLA as a way to logically treat low back pain, but responses did not differ for other aspects of treatment expectations [17]. In the current study, participants were asked to respond to the same statements after viewing two minute filmed demonstrations of the treatments. The investigators realize a better study design of measuring placebo effect would be to have the participants experience the treatments. This study, however, was conducted to provide pilot data on a topic that is under-researched, but vital to successfully conducting clinical trials of OMT. This represents an elementary step in identifying an appropriate placebo for OMT clinical trials.

Analysis revealed a significant difference in participant responses to the statement "I believe this treatment would allow me to get better quicker" after viewing the three treatments. Post hoc analysis demonstrated that participants responded more positively with the statement after viewing the High-Velocity Low-Amplitude treatment than after viewing the light touch treatment. There were no differences between responses after viewing the High-Velocity Low-Amplitude and ultrasound treatments or between ultrasound and light touch treatments.

There was also a significant difference between responses for the statement "I believe this treatment would make me more able to do the things I want to do." Again, post hoc analysis revealed a difference existed in participant responses after viewing the High-Velocity Low-Amplitude treatment and the light touch treatment such that participants responded more positively with the High-Velocity Low-Amplitude treatment. There were no other differences between responses to this statement. These results suggest the sub-therapeutic ultrasound might be a more suitable placebo than LT since there were significantly different expectations between the HVLA and the light touch treatments, but not between HVLA and ultrasound.

### Prior experience with manipulative treatment

The expectation of treatment and hence the placebo effect may change with a person's previous experience with manipulative treatment. In this study participants were asked if they had ever had osteopathic manipulative treatment or chiropractic treatment. Participants that had received manipulative treatment (non-naïve) were compared to participants who had never received manipulative treatment (naïve) on all four statements. There were differences between responses from naïve and non-naive participants for the statement "I believe this treatment would allow me to get better quicker," after viewing the ultrasound treatment. Participants who were naïve to manipulative treatment responded more favourably than those who were non-naïve, suggesting that naïve participants had a greater expectation of treatment after viewing the ultrasound treatment than did non-naïve participants. The difference in responses between naïve and non-naïve participants for the statement "This seems like a logical way to treat low back pain" approached significance, again suggesting that naïve participants may have had a greater expectation of treatment after viewing the ultrasound treatment. No differences were found between naïve and non-naïve participants after viewing the light touch treatment for any of the statements. These results suggest that differences in treatment expectations between naïve and non-naïve research participants can have important implications in selecting the appropriate placebo for manipulative treatment clinical trials. Research study participants' experience with manipulative treatment should be measured and controlled for through statistical analysis or controlled for in the study design.

### Limitations

There are several limitations to this current study. First, a convenience sample was utilized. Participates in this study were patients in a Family Medicine clinic and were asked to participate if they would be waiting at least 10 minutes before being seen by their physician. This recruitment process was used to ensure minimal interruption of clinic operations. The use of a convenience sample minimizes the generalizability of the study findings to the general population. Second, participants were recruited from a clinic associated with an osteopathic medical school. This may further limit generalizability because patients at an osteopathic medical school had self reported poorer health than the general population [19]. However, this clinic population is representative of subjects commonly used in OMT clinical research studies. Finally, study participants did not experience the different treatments. This study measured treatment expectations from watching a short demonstration of the treatments. Actually experiencing the treatments would provide additional stimuli to participants and potentially change their expectations.

### Future research

This study is only an initial step in identifying the best placebo for OMT clinical trials. Measuring expectations of benefits after viewing a two minute demonstration of a treatment cannot replace measuring expectations of benefits after actually receiving a treatment. Further research into the placebo effect, particularly for manipulative treatments, is needed. The most appropriate placebo for OMT clinical trials cannot be truly determined until a better understanding of treatment expectation, physician/patient interaction in osteopathic medicine, other factors involved in the placebo effect, and a patient's experience with manipulative treatment is achieved. A point of interest would be to determine if the amount of prior experience with manipulative treatment is correlated with treatment expectations. Additionally, prior experience with manipulative treatment may have different effects across different countries with variations in OMT practitioner training. The video demonstrations also do not control for the effect of touch or sound during the treatments. Actually applying the sub-therapeutic ultrasound on the skin with gel could potentially change treatment expectations. The presented results, however, provide an elementary analysis of potential placebo treatments in OMT clinical trials and provide the opportunity to seek funding for more definitive research studies. The next step is to research the way participants respond to these issues after actually experiencing OMT and placebo treatments.

## Conclusion

This pilot study presents valuable elementary information for selecting an appropriate placebo when conducting OMT clinical trials. Effect sizes between HVLA and two possible placebo treatments are provided. These effect sizes can be used to help determine sample size estimations for future projects involving either a light touch or sub-therapeutic ultrasound placebo group as compared to an HVLA treatment group. Results of this pilot study suggest that expectations of the sub-therapeutic ultrasound placebo treatment are more similar to expectations of the HVLA treatment. Thus, using a sub-therapeutic ultrasound placebo may allow for a more optimal opportunity to identify a true treatment versus placebo effect. Additionally, it is important to control for prior experience with the various treatments, as those who are not naïve to manipulative treatment may have greater treatment expectations. The authors recommend either excluding subjects who have had OMT or chiropractic treatment or at least using statistical analyses to control for potential effects.

## Competing interests

The author(s) declare that they have no competing interests.

## Authors' contributions

KGF participated in the design of the project, recruited subjects, collected data, performed the statistical analysis, and drafted the manuscript. TS participated in the design of the project, participated in developing the data collection forms, and participated in editing and finalizing the manuscript. STS participated in the design of the study, acquired resources, participated in developing the video taped treatments, and participated in editing and finalizing the manuscript. All authors read and approved the final manuscript.
